# The improvement of heat transfer using Co/SiO_2_ spiral structured catalyst for green diesel production by Fischer–Tropsch synthesis

**DOI:** 10.1038/s41598-024-70503-6

**Published:** 2024-08-26

**Authors:** Pronprom Aurud, Atthapon Srifa, Wanida Koo-Amornpattana, Suttichai Assabumrungrat, Suwimol Wongsakulphasatch, Choji Fukuhara, Sakhon Ratchahat

**Affiliations:** 1 Department of Chemical Engineering, Faculty of Engineering, Mahidol University, Nakhon Pathom, 73170 Thailand; 2 Center of Excellence in Catalysis and Catalytic Reaction Engineering, Department of Chemical Engineering, Faculty of Engineering, Chulalongkorn University, Bangkok, 10330 Thailand; 3 Department of Chemical Engineering, Faculty of Engineering, King Mongkut’s University of Technology North Bangkok, Bangkok, 10800 Thailand; 4 Department of Applied Chemistry and Biochemical Engineering, Graduate School of Engineering, Shizuoka University, Shizuoka, 432-8561 Japan

**Keywords:** Fischer–Tropsch synthesis, Cobalt structured catalyst, Heat transfer, Green diesel, Renewable energy, Chemical engineering, Materials for energy and catalysis

## Abstract

In this study, the improvement of heat transfer was applied to eliminate hotspots of a highly exothermic reaction, Fischer–Tropsch synthesis (FTS), by means of two facile methods: (I) adding high thermal conductive materials media diluted in catalysts (SiC and Al chips), and (II) using structured reactors equipped with well-designed structured catalysts with advantages of heat dissipation/removal. The 20%Co/SiO_2_ catalyst powder prepared by simple impregnation was employed for constructing structured catalysts and granular packed bed catalysts. The structured catalyst was prepared by coating method of Co/SiO_2_ slurry on an aluminum spiral and plate substrate. The catalytic performance of as-prepared catalysts was then tested for FTS in a fixed-bed reactor at 210–230 °C, 20 bar. Both gaseous and liquid products were collected and analyzed. The heat transfer improvement of packed bed catalytic system and structured catalytic system were compared and discussed. As a result, the structured catalytic system with spiral structured catalyst can provide the best improvement of heat/mass transfer, resulting in enhanced diesel selectivity, though the oil production rate was unsatisfactory. Meanwhile, among the packed bed catalytic systems, SiC media possessed the best heat removal material, producing the highest oil yield. In addition, the fresh and spent catalysts were analyzed by several techniques including TEM, SEM, XRD, BET, ICP-OES, H_2_–TPR, and TGA to relate the physicochemical properties of the prepared catalysts and its FTS performance.

## Introduction

The consumption of fuel and chemical products produced from petroleum crude oil or fossil fuel is continuously increasing, while the fossil-based resource is sharply decreasing. The alternative options such as renewable energy have been receiving great attention because of the quality of synthetic liquid fuels which are high cetane number and release of noxious chemicals lower than conventional processes. Furthermore, these synthetic liquid fuels have the potential to serve various purposes, the same as fossil fuels for stationary power such as compressed natural gas (CNG) and liquefied petroleum gas (LPG) and for transportation (gasoline, diesel). Numerous methods exist for producing liquid fuels, such as gas-to-liquid (GTL), biomass-to-liquid (BTL), and coal-to-liquid (CTL). Among GTL technologies, Fischer–Tropsch (FT) synthesis stands out, as it transforms synthesis gas (mixture of CO and H_2_) into liquid hydrocarbon fuels using heterogeneous catalysts at atmospheric or moderate hundreds of degrees^1–3^. The product from this process is widely distributed such as light hydrocarbons e.g., compressed natural gas (C_1_–C_2_), pressures and temperatures reaching liquefied petroleum gas (C_3_–C_4_), premium middle distillate cuts e.g., gasoline (C_5_–C_11_), diesel (C_12_–C_20_), jet fuel (C_9_–C_16_) and heavier cuts e.g., solid wax (C_20+_)^4,5^. The Co-based catalysts are widely used at the industrial scale because of their high activity, good resistance to deactivation, flexibility of operation, and high selectivity to linear long-chain hydrocarbons. The catalytic activity and the structures of catalysts depend on particle size, morphology, and crystallographic phase of the nanoparticles, which influence FT reaction mechanism^6^. The Fischer–Tropsch synthesis process involves two reaction stages for producing liquid fuels. The first stage, the FT reaction/FT functionality in Eq. (1), the growth of the straight-chain hydrocarbon, which can be paraffins, olefins and aromatics. Most of the oxygen from CO dissociation has a water–gas shift (WGS) reaction in Eq. (2) produces water and carbon dioxide as the main products. Next, the second stage, hydrocracking or hydrotreating to separate and upgrade the liquid hydrocarbon fuels from the previous step by isomerization to produce the branched hydrocarbons^7^.

1 $${\text{CO}} + 2{\text{H}}_{2} \to ( - {\text{CH}}_{2} - ) + {\text{H}}_{2} {\text{O}}\quad \Delta {\text{H}}_{{298{\text{K}}}} = - \left( {165 - 180} \right) \;{\text{kJ/mol}}$$

2 $${\text{CO}} + {\text{H}}_{2} {\text{O}} \to {\text{CO}}_{2} + {\text{H}}_{2} \quad \Delta {\text{H}}_{{298{\text{K}}}} = - 41 {\text{kJ/mol}}$$

Because FT reaction is exothermic reaction, hotspots can easily form on the catalyst surface impacted the performance of FT synthesis and causing the production of lighter hydrocarbon products instead. Thus, improving heat transfer development is one challenge of Fischer–Tropsch synthesis^8,9^. To enhance heat transfer properties, it can be placed in two easy ways: (I) adding high thermal conductive materials diluted in catalysts, for example, silicon carbide (SiC) and aluminum, and (II) using a structured reactor equipped with well-designed structured catalysts. Derevish et al.^10^ reported that the temperature at the center of the catalyst granule was higher than that at the surface. Variations in the thermal conductivity of the catalyst granule could influence the temperature distribution within it, resulting in activity instability and an uneven distribution of hydrocarbon products. Many researchers have attempted to address this problem. Da Wang et al. believed that the main cause of this problem is the thermal conductive property of the catalyst which affected the hydrocarbon product distribution. Thus, they had modified the thermal conductive property by adding high thermal conductive aluminum powder in different proportions to catalysts^10^. The other way to enhance heat transfer properties is a structured reactor with a structured catalyst. This concept focuses on effective heat management by utilizing a highly conductive substrate. Additionally, with laminar flow hydrodynamics, the reactant gases can fully access the catalytic surface, resulting in reduced pressure drop and minimized mass transfer limitations^11^. In short, there are many pathways to produce liquid fuels from renewable resources, and FT synthesis is one of promising technologies. The challenge is commercial scale production and heat management due to the exothermic reaction of FT synthesis reaction. Therefore, the aim of this research is to develop the cobalt based structured catalyst for green diesel production and find the optimum condition for green diesel production by FT synthesis.

## Experimental

### Preparation of Co/SiO_2_ catalyst

**Fig. 1:**
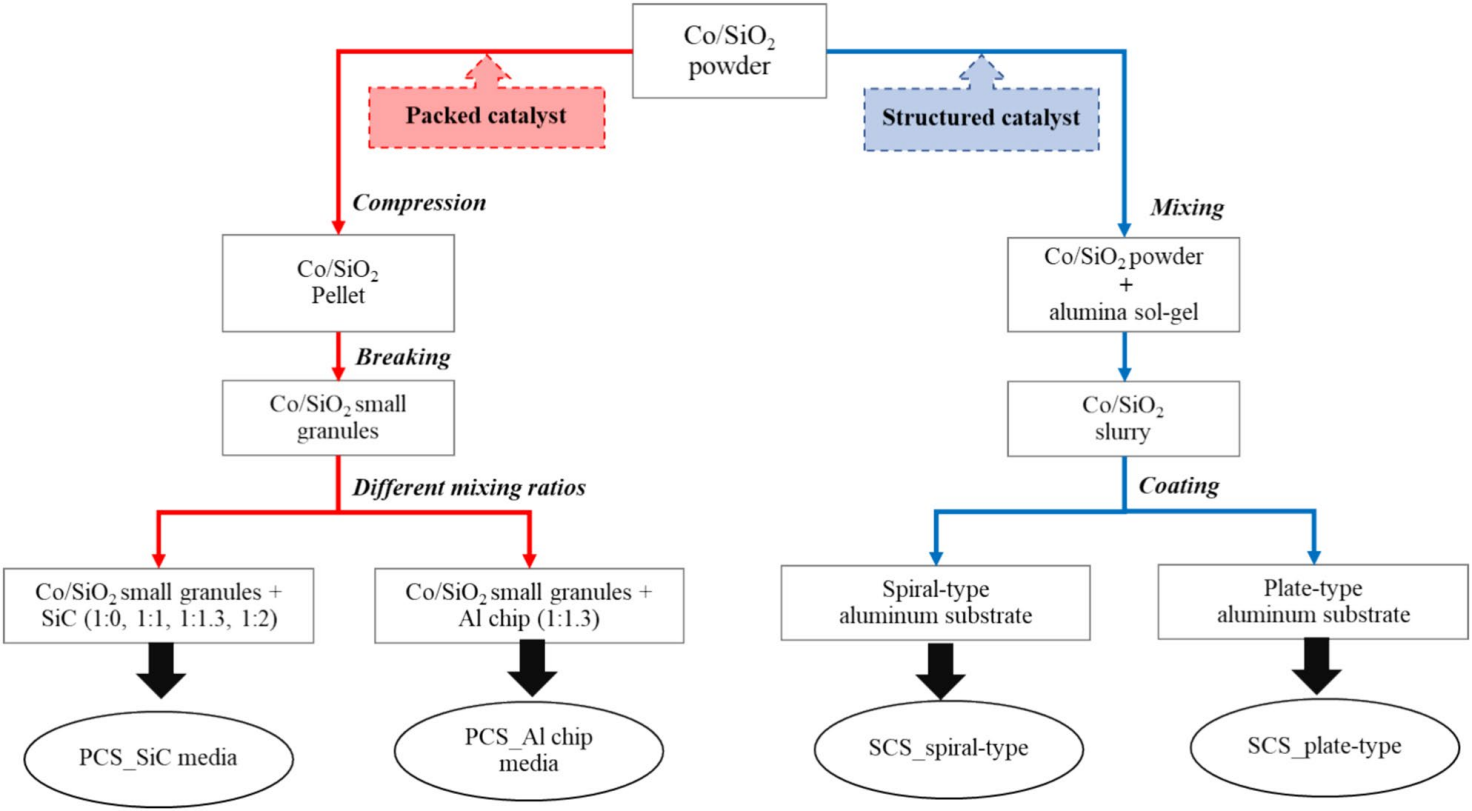
Schematic of experiment cases for the packed bed catalysts and the structured catalysts.

Co/SiO_2_ catalyst with 20%wt Co loading was prepared by impregnation method. A cobalt nitrate hexahydrate (Co(NO_3_)_2_⋅6H_2_O, 98% purity, Carlo Erba) as a cobalt precursor was dissolved in de-ionized (DI) water and mixed with SiO_2_ powder (fumed silica, 200 m^2^/g, Sigma-Aldrich,) as a catalyst support. Then, the mixture evaporated at 80 °C under stirring condition to dryness. The dried sample was further dried in oven overnight at 80 °C. Afterward, the dried catalyst was calcined in air at 400 °C for 6 h with ramp rate of 10 °C/min. The obtained catalyst powder was used for preparing the structured catalysts and the packed bed catalysts. The experiments were divided into 4 cases as shown in Fig. 1.

### Preparation of packed bed catalysts and structured catalysts

**Fig. 2:**
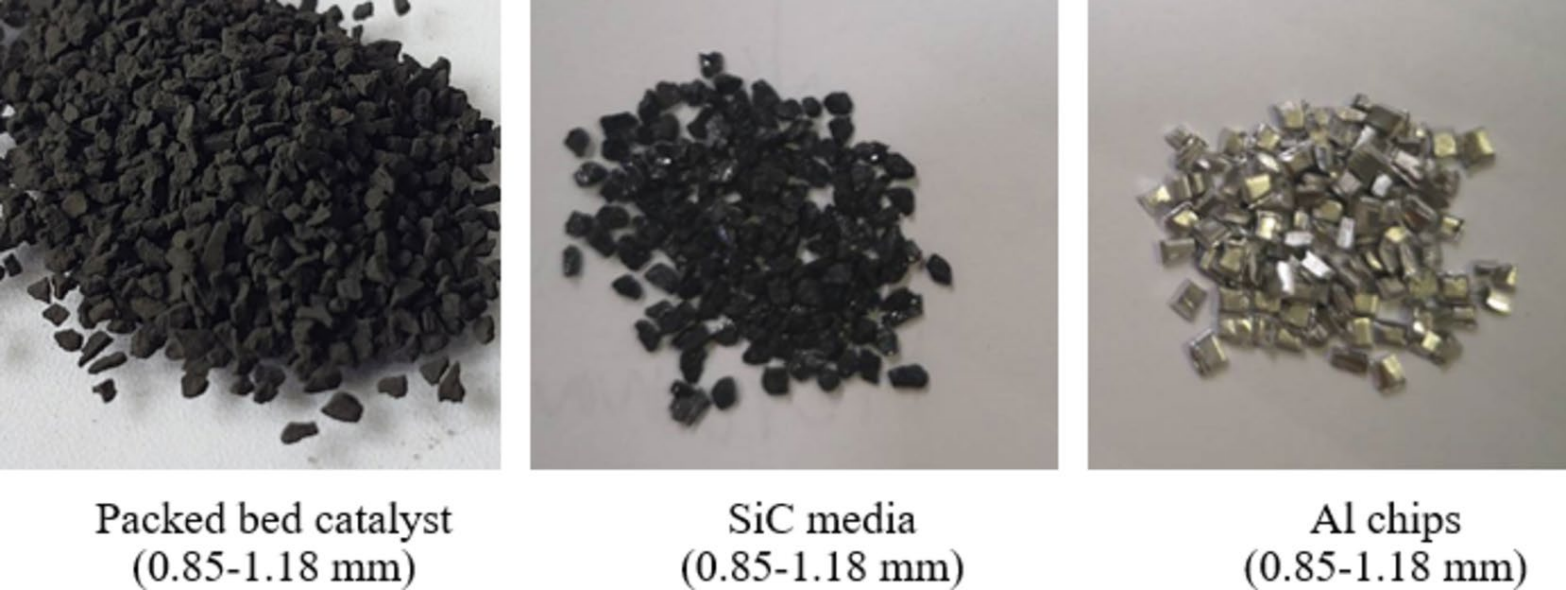
Packed bed catalytic system (PCS) and high thermal conductive materials (SiC, Al chips).

The packed bed catalyst was used in the packed bed catalytic system (PCS). The making of packed bed catalyst was prepared by pressing 2 g of Co/SiO_2_ catalyst powder with hydraulic hand pump at 40 MPa for 10 min, then breaking and sieving to catalyst granule with size of 0.85–1.18 mm as shown in Fig. 2. Then, the catalyst granules were manually mixed with high thermal conductive materials including silicon carbides (0.85–1.18 mm, SiC) and aluminum cutting chips (Al chips).

**Fig. 3:**
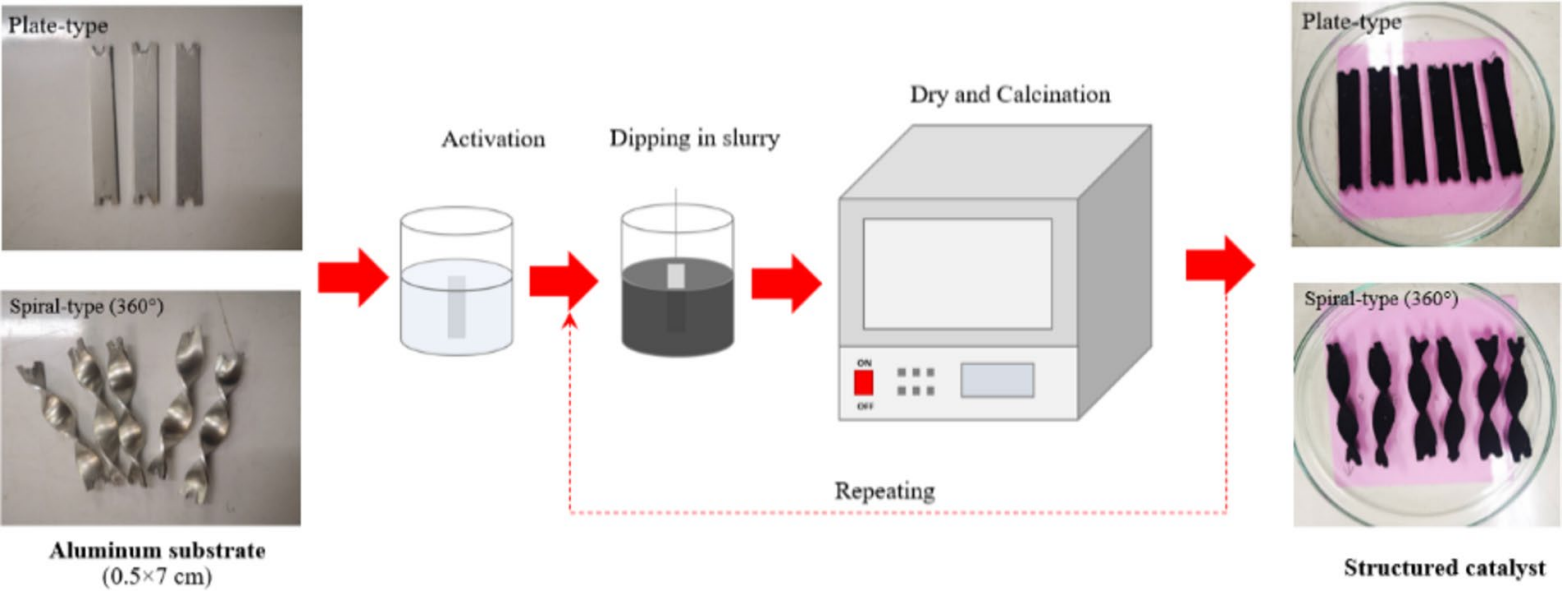
Procedure for preparation of structured catalysts (plate type and spiral type).

In the structured catalytic system (SCS), the as-prepared Co/SiO_2_ catalyst powder was used for preparation of the structured catalysts by coating method. Figure 3 shows a procedure for preparation of plate/spiral Co/SiO_2_ structured catalysts. The rectangle shape of aluminum substrate with a length of 50 mm and a width of 7 mm was used. The width was slightly smaller than the diameter of reactor (10 mm) to provide a clearance for packing and to dedicate for the thickness of catalyst coating. In the case of spiral-type, the aluminum substrate was fabricated with a twist angle of 360°. Before the coating, the aluminum substrate was cleaned with NaOH solution (0.8 N), and then activated by HCl (2.8 N) to obtain a uniformly rough surface^12^. After the cleaning/activating step, the aluminum substrate was dipped into a slurry of Co/SiO_2_ catalyst. The slurry was prepared by Co/SiO_2_ catalyst powder in alumina sol–gel as a binder with weight ratio of sol–gel:catalyst powder of 30 g:10 g. The alumina sol–gel was prepared from solution of aluminum tri-isopropoxide, nitric acid, formaldehyde, and water (Al(OCH(CH_3_)_2_)_3_): 24 g, HNO_3_: 12 mL, HCHO: 8 mL, H_2_O: 160 mL)^13^. In every layer of coating, the sample was blown to be dried before the next layer was coated. The coating was done several times to obtain a certain amount of catalyst (1 g/piece). The coated catalysts were then calcined in air at 100 °C for 30 min and at 500 °C for 2 h. The spiral-type substrate was called a spiral structured catalyst and plate-type substrate was called a plate structured catalyst.

### Catalytic performance test by Fischer–Tropsch synthesis

**Fig. 4:**
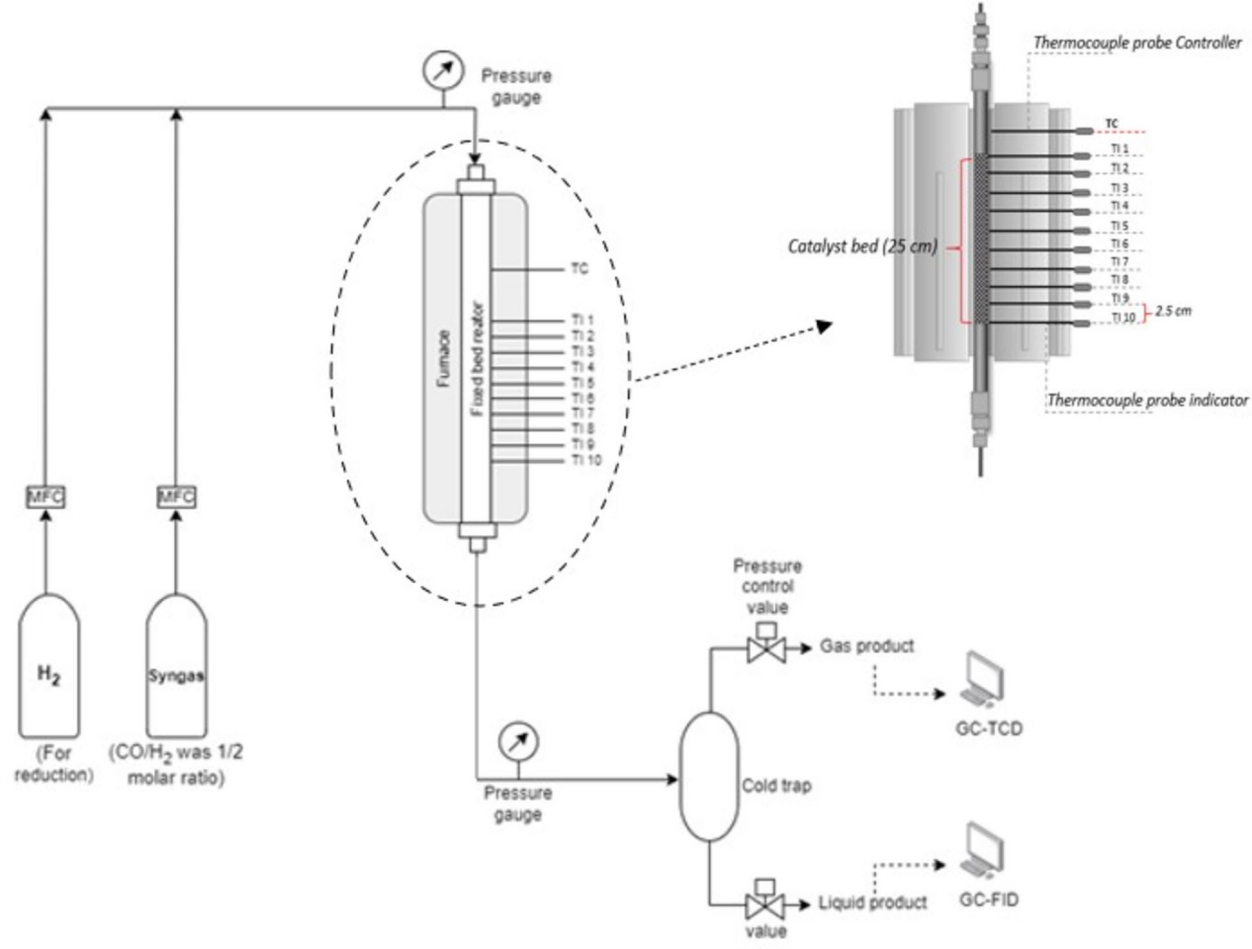
Schematic of the experimental setup for Fischer–Tropsch synthesis.

Figure 4 shows the experimental setup for the Fischer–Tropsch synthesis (FTS) reaction used in this work. The FTS performance of as-prepared catalysts was evaluated by using a continuous fixed-bed reactor with length of 700 mm and an internal diameter (ID) approximately 10 mm (made of 316 SS, UTSAKAN. CO., LTD.). Typically, 5 g of Co/SiO_2_ catalyst was used for granule testing and for structured catalsyts. The catalyst was activated by H_2_ reduction at 400 °C for 3 h with heating rate of 10 °C/min under pure H_2_ flow of 100 ml/min. After the reduction completed, the reactor was then cooled down to room temperature, and a mixture of reaction gas which is syngas (33%CO/H_2_, moisture content ≤ 5 ppm, United Industrial Gas Co., Ltd.) with CO/H_2_ ratio of 1/2 was continuously fed into the reactor from room temperature at feed flow rate of 100 ml/min and gauge pressure of 20 bar. The reactor was then heated up to the reaction temperature with ramp rate of 10 °C/min. The suitable reaction temperature was studied in the range of 210, 220, and 230 °C. For each experiment run, the FTS process was continuously tested for 8 h. The outlet gas was collected and analyzed by gas chromatography every 1 h (GC2014-TCD, Shimadzu), while the liquid products were collected at once after testing for 8 h.

### Packing of structured catalytic system (SCS) in the FTS reactor

**Fig. 5:**
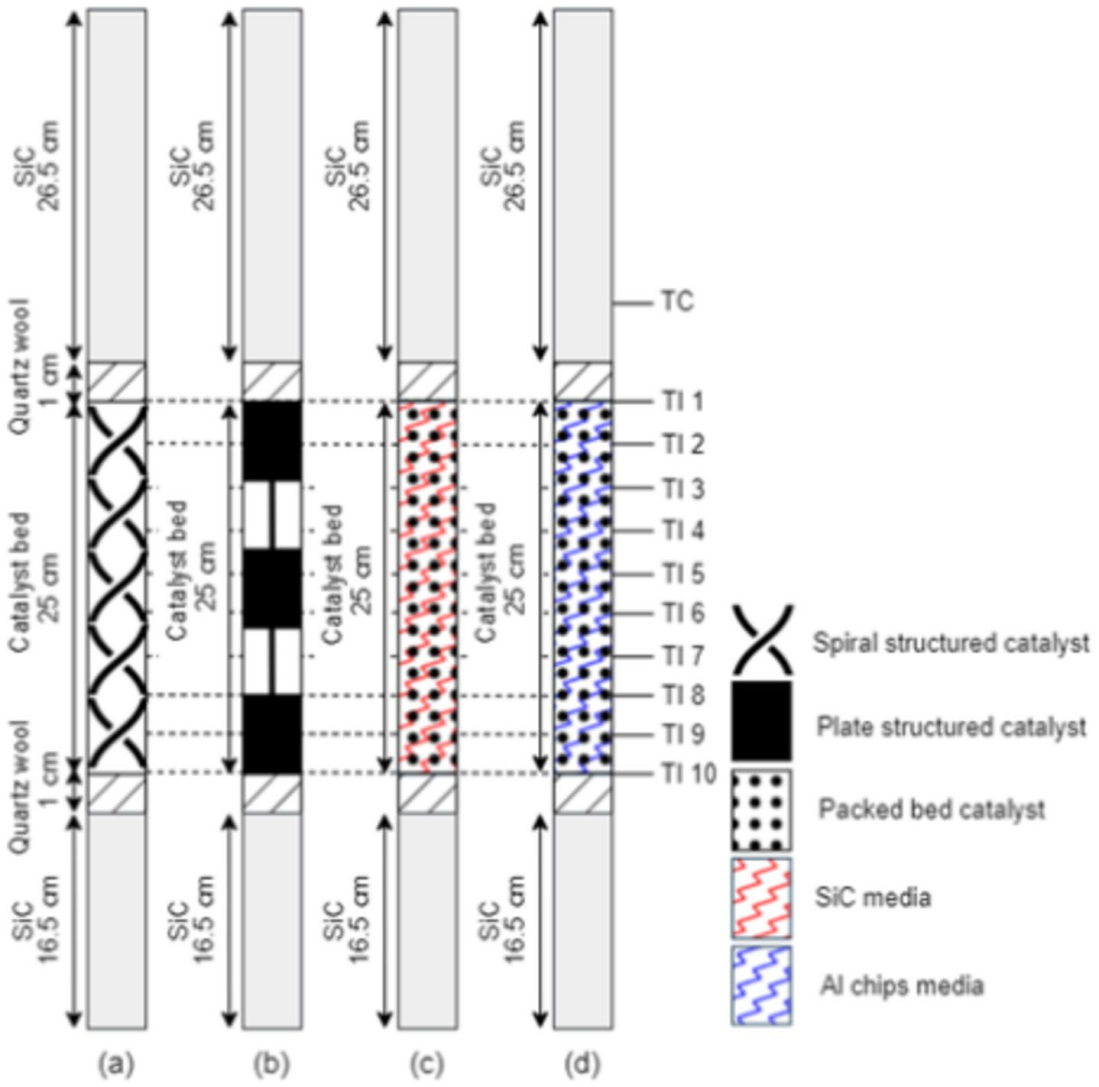
The schematic packing of structured catalytic system and packed bed catalytic system: (**a**) structured catalytic system with spiral-type structured catalyst (SCS_spiral-type), (**b**) structured catalytic system with plate-type structured catalyst (SCS_plate-type), (**c**) packed bed catalytic system diluted with SiC pellets (PCS_SiC), and (**d**) packed bed catalytic system diluted with Al chips (PCS_Al chips).

The structured catalyst with 5 pieces (total catalyst bed length of 25 cm) was carefully loaded into the fixed-bed reactor as schematically shown in Fig. 5a,b. Henceforth, there are two types of structured catalytic system: structured catalytic system equipped with spiral structured catalyst named SCS spiral-type and structured catalytic system equipped with the plate structured catalyst named SCS_plate-type. At the inlet of the reactor, SiC pellets (diameter size 0.85–1.18 mm) were loaded till the first temperature indicator (TI 1) level and quartz wool of 10 mm. Similarly, at the outlet of the reactor, SiC pellets were loaded till the end of the reactor.

### Packing of packed bed catalytic system (PCS) in the FTS reactor

**Fig. 6:**
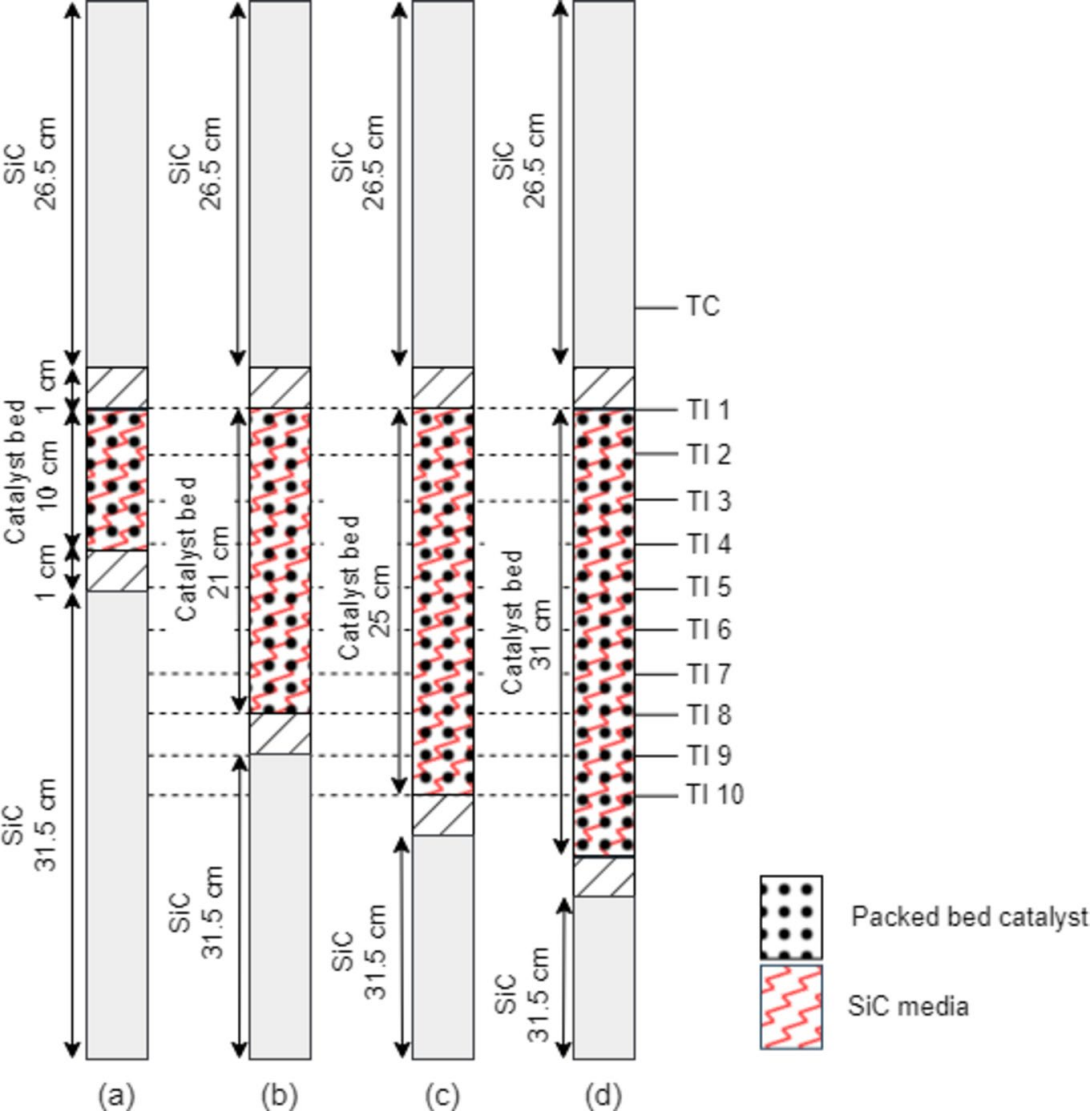
The schematic packing of packed bed catalytic system diluted with SiC pellets (PCS_SiC) in different dilution ratios (**a**) 1:0, (**b**) 1:1.0, (**c**) 1:1.3, and (**d**) 1:2.0.

The packed bed catalytic system was loading with 5 g of packed bed catalyst diluted with some amount of high thermal conductive material media. In this research, we used SiC pellets and Al chips (diameter size of 0.85–1.18 mm). This packed bed catalytic system varies the volumetric ratio of the high thermal conductive material media and catalyst. The volumetric ratio of the catalyst:SiC pellets is 1:0 (No dilution), 1:1.0, 1:1.3, and 1:2.0, while the catalyst: Al chip is 1:1.3. The volumetric fraction of 1:1.3 was a form of a catalyst bed length 25 cm, equally to the catalyst bed length of structured catalytic system. The schematic packing of packed bed catalytic system is shown in Figs. 5c, d, and 6. Henceforth, the packed bed catalytic system diluted with SiC named PCS_SiC media was also diluted with Al chips named PCS_Al chips.

### Catalyst characterization

The catalytic properties of catalysts were characterized by various techniques. Physical properties such as surface area, pore volume, and pore diameter of the catalyst were analyzed by Brunauer–Emmett–Teller (BET) and the BJH (Barrett, Joyner, and Halenda) method. The morphology image of fresh and spent catalysts such as cobalt dispersion on support, and particle size distribution was obtained from Scanning Electron Microscope (SEM) and Transmission Electron Microscope (TEM). X-ray diffraction (XRD) was used for a material crystallographic structure before and after the experiment. Thermogravimetric (TGA) is used to investigate carbon deposition on the catalyst to find the appropriate calcined temperature and to determine the reduction of weight of spent catalysts. The temperature was increased from room temperature to 1000 °C at a heating rate of 10 °C/min with oxygen flow of 50 ml/min. The catalyst reducibility was analyzed by hydrogen temperature programmed reduction (H_2_-TPR).

### Product analysis

The gas and liquid products were separated for analysis. The gas product was analysed during experiment every 1 h by gas chromatography with TCD detector. For the liquid product, the oil phase products were separated from the water before analysis offline by gas chromatography with FID detector. CO conversion was calculated from the difference of CO molar flowrates at the inlet and outlet, as shown in Eq. (3):

3 $$X_{CO} \;\left[ \% \right] = \frac{{F_{CO,in} - F_{CO,out} }}{{F_{CO,in} }} \times 100$$

where $F_{CO,in}$ and $F_{CO,out}$ are the molar flowrates of CO inlet and outlet, respectively.

The selectivity is reported in two types: the molar selectivity to each product based on carbon number; the mass selectivity of gasoline range (C_5–11_) and diesel range (C_12–20_) in the oil phase^14^ as shown in Eqs. (4–6).

4 $$S_{C1 - 4} \;\left[ \% \right] = \frac{{n_{1 - 4} F_{C1 - 4} }}{{F_{CO}^{in} - F_{CO}^{out} }} \times 100$$

5 $$S_{C5 + } \;\left[ \% \right] = \left( {1 - \mathop \sum \limits_{i} \frac{{n_{i} F_{C1 - 4} }}{{F_{CO}^{in} - F_{CO}^{out} }}} \right) \times 100$$

6 $$S_{diesel} \;\left[ \% \right] = \left( {\frac{Area \;of\; diesel \;product}{{Area \;of\; total\; oil\; product}}} \right) \times 100$$

7 $$Yield\; _{i} \left[ \% \right] = F_{CO}^{0} \cdot \chi_{CO} \left( {S_{i} } \right)$$

The molar selectivity to CO_2_ and C_1_-C_4_ species was calculated by Eq. (4). $F_{ci}$ is the molar flowrate of species C_i_. $n_{i}$ is the number of carbon atoms in each species. The C_5+_ selectivity was calculated by Eq. (5). The quantitative of the diesel range product was calculated by Eq. (6), according to non-practical direct quantification with traditional calibrated peak area procedure. The yield of each species can be calculated from Eq. (7), where $F_{CO}^{0}$ is inlet CO molar flow rate, $\chi_{CO}$ the CO conversion and *S*_*i*_ is selectivity of hydrocarbon species i. Several researchers use the corresponding peak area of products to determine the selectivity of desired products^15^. Oil phase product selectivity in this research based on the corresponding alkane peaks areas from C_8_–C_20_ calibration that performed C_8_–C_20_ Alkane standard solution (Sigma-Aldrich). The percentage of peak area of diesel range product to the total peak areas was obtained from GC-FID chromatogram.

## Results and discussion

### Characterization of Co/SiO_2_ catalysts

**Fig. 7:**
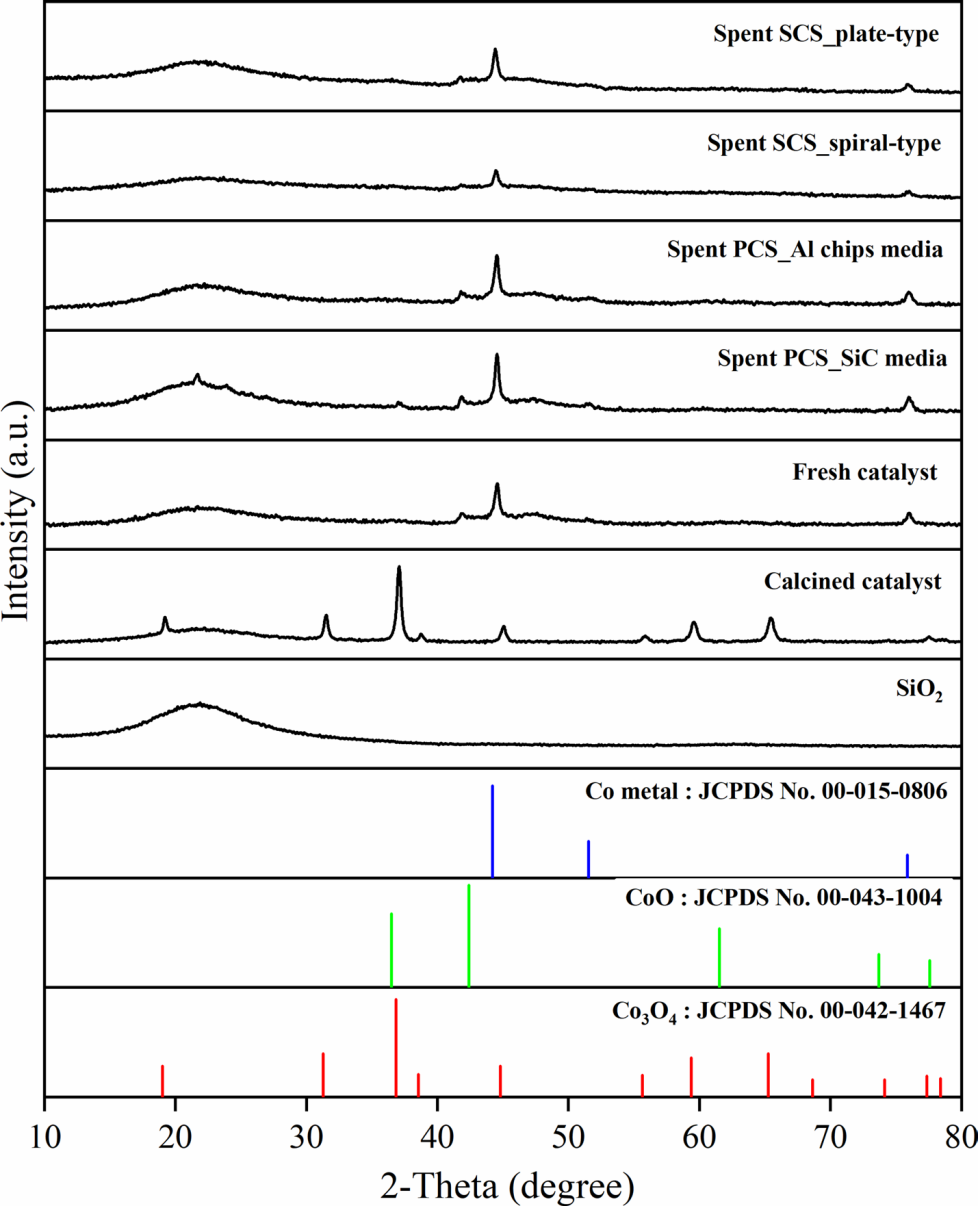
XRD patterns of SiO_2_ support, calcined, fresh reduced, and spent Co/SiO_2_ catalysts.

Figure 7 shows X-ray diffraction patterns of the as-prepared Co/SiO_2_ catalysts including the calcined catalyst, the reduced catalyst (noted as the fresh catalyst) and the spent structured catalysts. It was found that all the catalyst samples presented a broad peak at around 21.1°, corresponding to amorphous SiO_2_ phase. The characteristic peaks of Co metal (Co^0^) and CoO were observed in the fresh catalysts after the reduction process. This indicated two reduction steps of Co_3_O_4_ to CoO and then to Co metal. The sharp peak of Co metal phase was clearly found in the spent PCS catalyst. This implied a larger particle size of Co metal on catalyst was obtained, compared with the spent SCS catalyst. The results related to the crystalline size of Co metal as listed in Table 1. The spent PCS_SiC catalyst exhibited a larger crystalline size of Co metal. It was speculated that metal sintering occurred through the application of heat and pressure reactions^16,17^. The metal sintering process has extremely high potential to occur due to the highly exothermic reaction of FTS. Furthermore, high water vapor provided by the secondary product accelerates the sintering process. The characteristic CoO peak at around 36.5° was evidently detected in the spent PCS_SiC catalyst, indicating the re-oxidation of cobalt active sites to cobalt oxide.

**Table 1:** Summary of physicochemical properties of support, fresh, and spent catalysts.

Catalyst	S_BET_^a^ (m^2^/g)	Total pore volume^a^ (cm^3^/g)	Pore diameter^a^ (nm)	Crystallite size^b^ (nm)			Composition^c^ (wt%)		
				Co_3_O_4_	CoO	Co^0^	Co	Al	Si
SiO_2_ support	164.2	1.2850	33.8	–	–	–	–	–	–
Fresh SCS catalyst	184.1	1.2570	34.3	–	–	–	19.50	6.02	48.54
Fresh PCS catalyst	143.6	0.6360	18.9	–	14.0	22.8	26.05	0.40	21.84
Spent SCS_spiral-type catalyst	86.5	0.5640	18.9	–	–	23.8	20.90	5.76	86.64
Spent SCS_plate-type catalyst	86.5	0.7060	34.9	–	–	27.2	17.35	4.70	25.91
Spent PCS_SiC media catalyst	95.6	0.5120	18.9	–	–	28.2	21.19	1.51	52.39
Spent PCS_Al chips media catalyst	103.5	0.5480	15.6	–	–	24.2	21.17	0.39	43.33

^a^Determined by BET method, ^b^Determined by XRD results, ^c^Determined by ICP-OES technique.

**Fig. 8:**
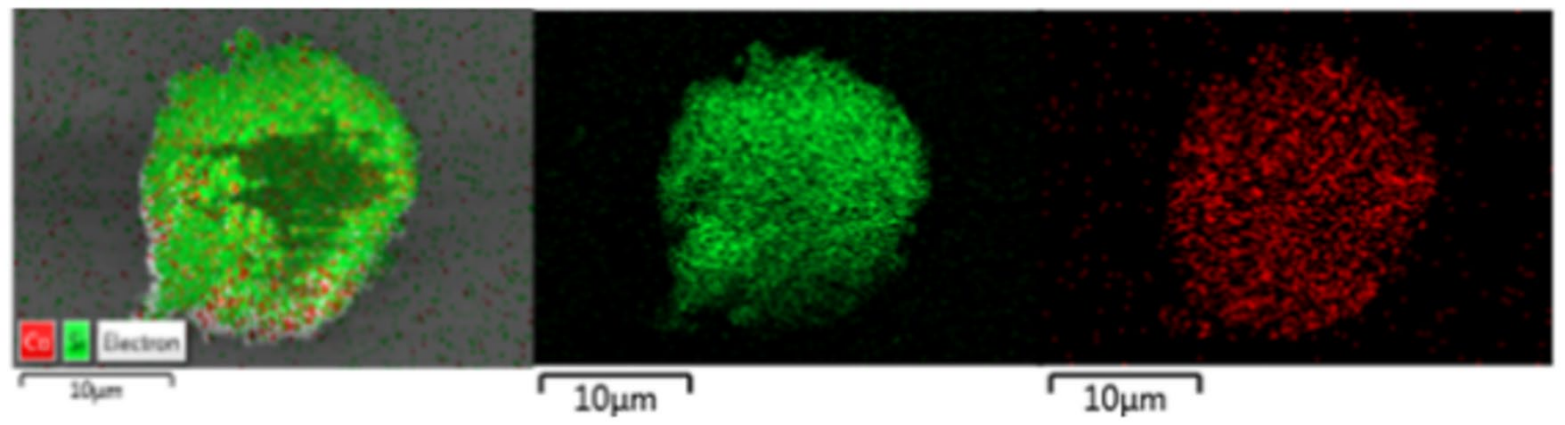
SEM–EDS morphology and element mapping of Co (red) and Si (green) of fresh catalyst powder.

**Fig. 9:**
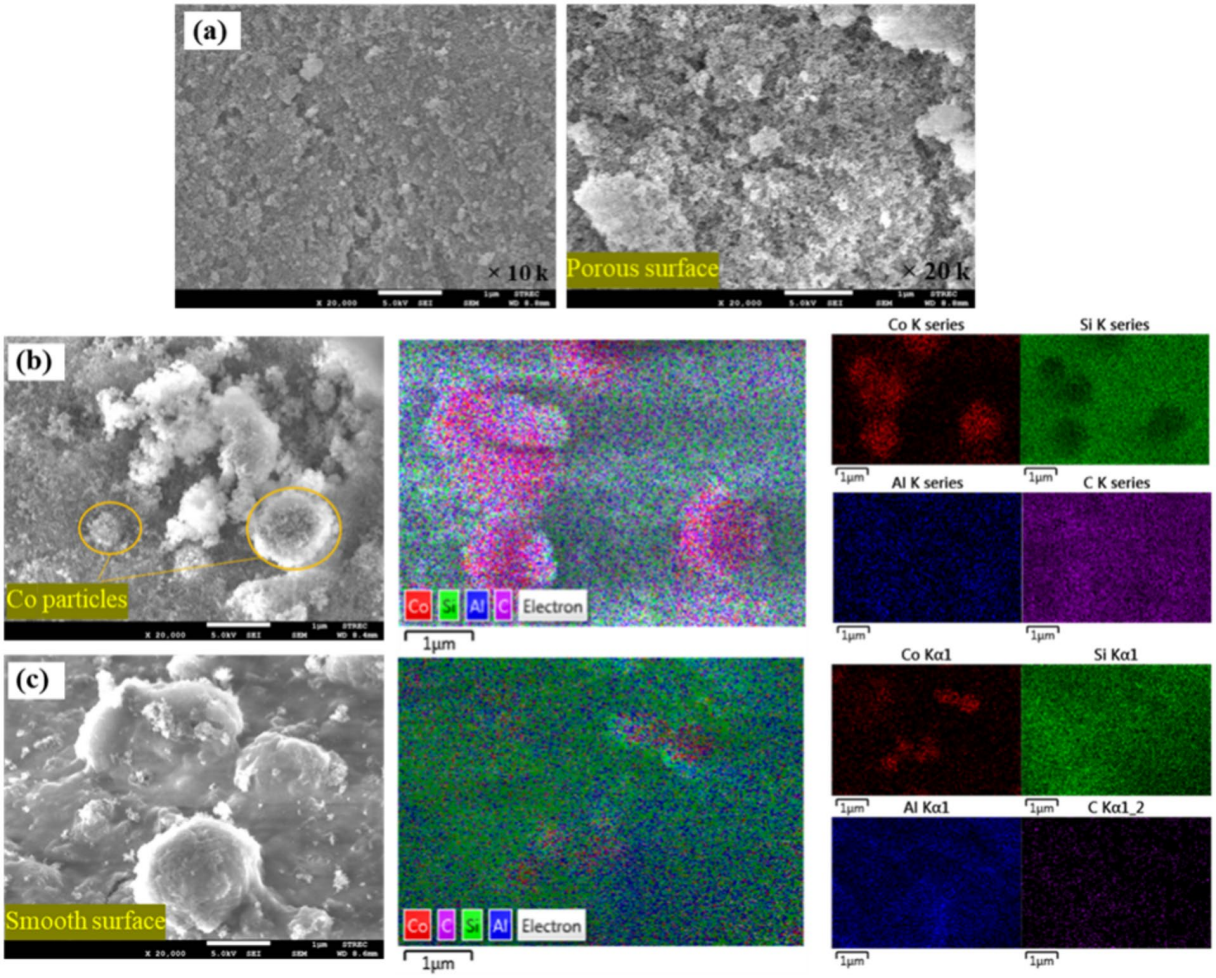
SEM–EDS morphology of the fresh catalysts (5 kV, ×20,000): (**a**) SiO_2_ support, (**b**) fresh PCS catalyst and (**c**) fresh SCS catalyst.

**Fig. 10:**
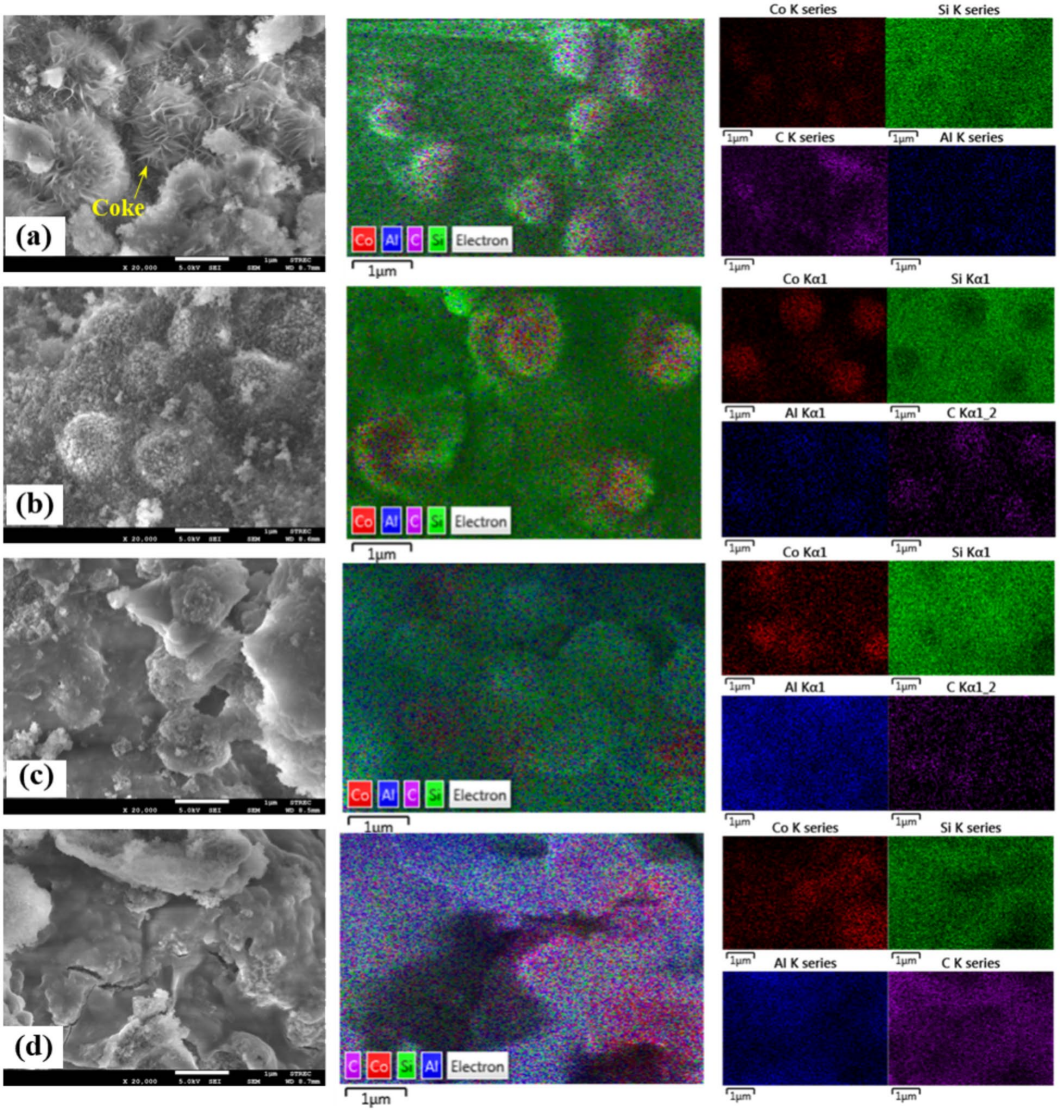
SEM–EDS morphology of the spent catalysts (5 kV, ×20,000): (**a**) spent PCS_SiC media catalyst, (**b**) spent PCS_Al chips media catalyst, (**c**) spent SCS_spiral-type catalyst and (**d**) spent SCS_plate-type catalyst.

Figures 8, 9 and 10 show SEM micrographs of fresh and spent Co/SiO_2_ catalyst. The SiO_2_ support showed a porous surface. After loading and reducing cobalt, the cobalt element was well dispersed on SiO_2_ support (see in Fig. 8). For both fresh structured catalyst and packed bed catalyst, it appeared that the cobalt metal on the fresh PCS catalyst is more effectively dispersed on the support than on the SCS catalyst (see in Fig. 9). The fresh PSC catalyst and the spent PSC catalyst displayed different morphology. The spent PSC_SiC and Al chip catalysts obviously showed carbon deposition on their surface, especially the spent PSC_SiC catalyst (see in Fig. 10). After the catalytic reaction, the structured catalysts maintained a similar morphology. It may be a sol–gel coating that has been applied to the catalyst, obviously detected by EDS mapping. It was remarked that the stabilized cobalt particles on the substrate inhibited coke formation on surface.

**Fig. 11:**
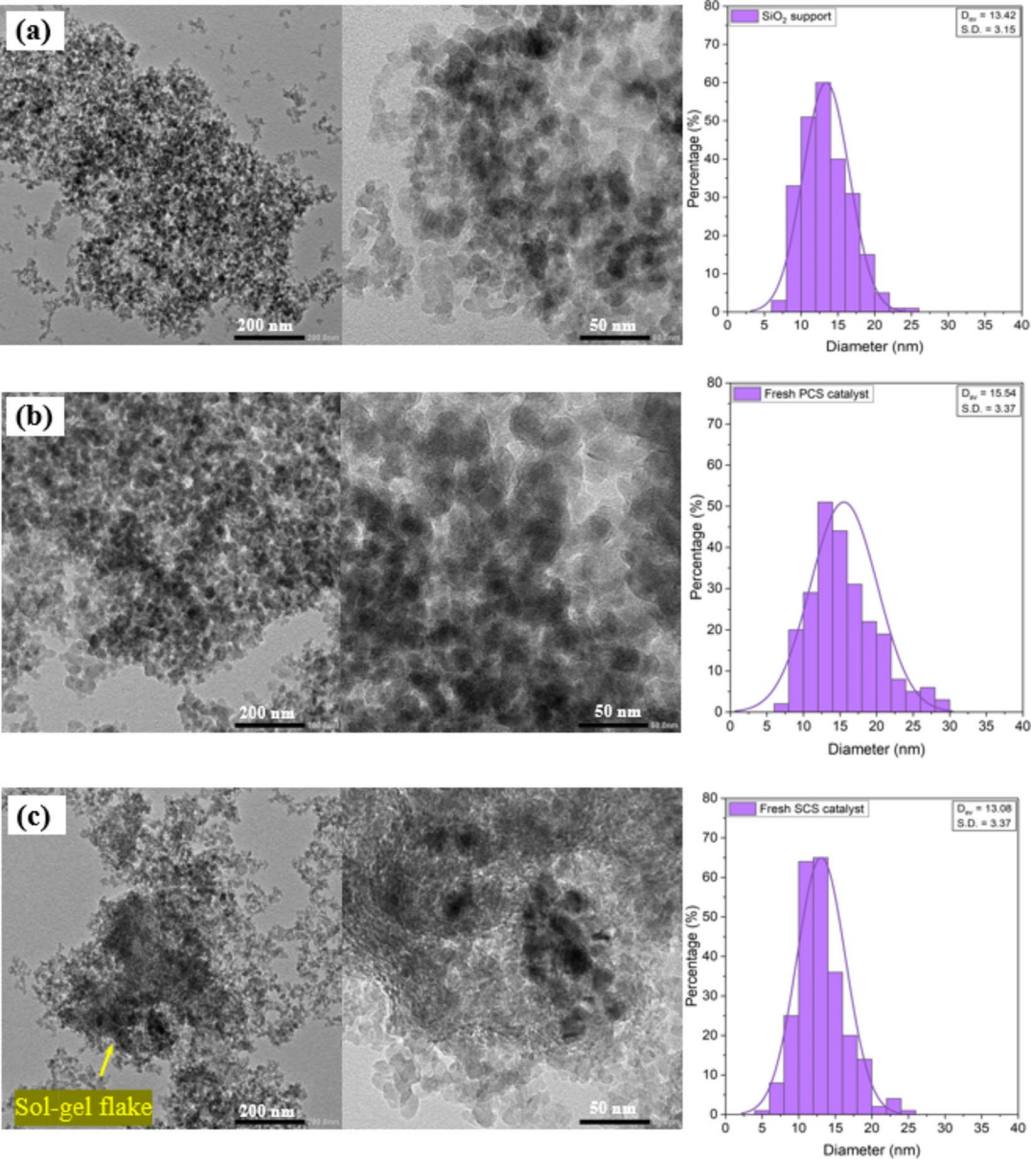
TEM images and particles size distribution of fresh catalysts: (**a**) SiO_2_ support, (**b**) fresh PCS catalyst and (**c**) fresh SCS catalyst.

Figure 11 shows TEM images of SiO_2_ support and fresh catalysts. The shape of particles for all samples was rather spherical. The TEM images of SiO_2_ support, and the PCS catalyst were slightly similar. It was indicated that cobalt particles were well dispersed on SiO_2_. However, the particle size of the fresh PCS catalyst varied and exhibited a wide distribution of sizes. Meanwhile, the fresh SCS catalyst displayed a narrower range of particle size distribution. The structured catalyst clearly exhibited the sol–gel flake and alumina sol–gel texture on its surface.

**Fig. 12:**
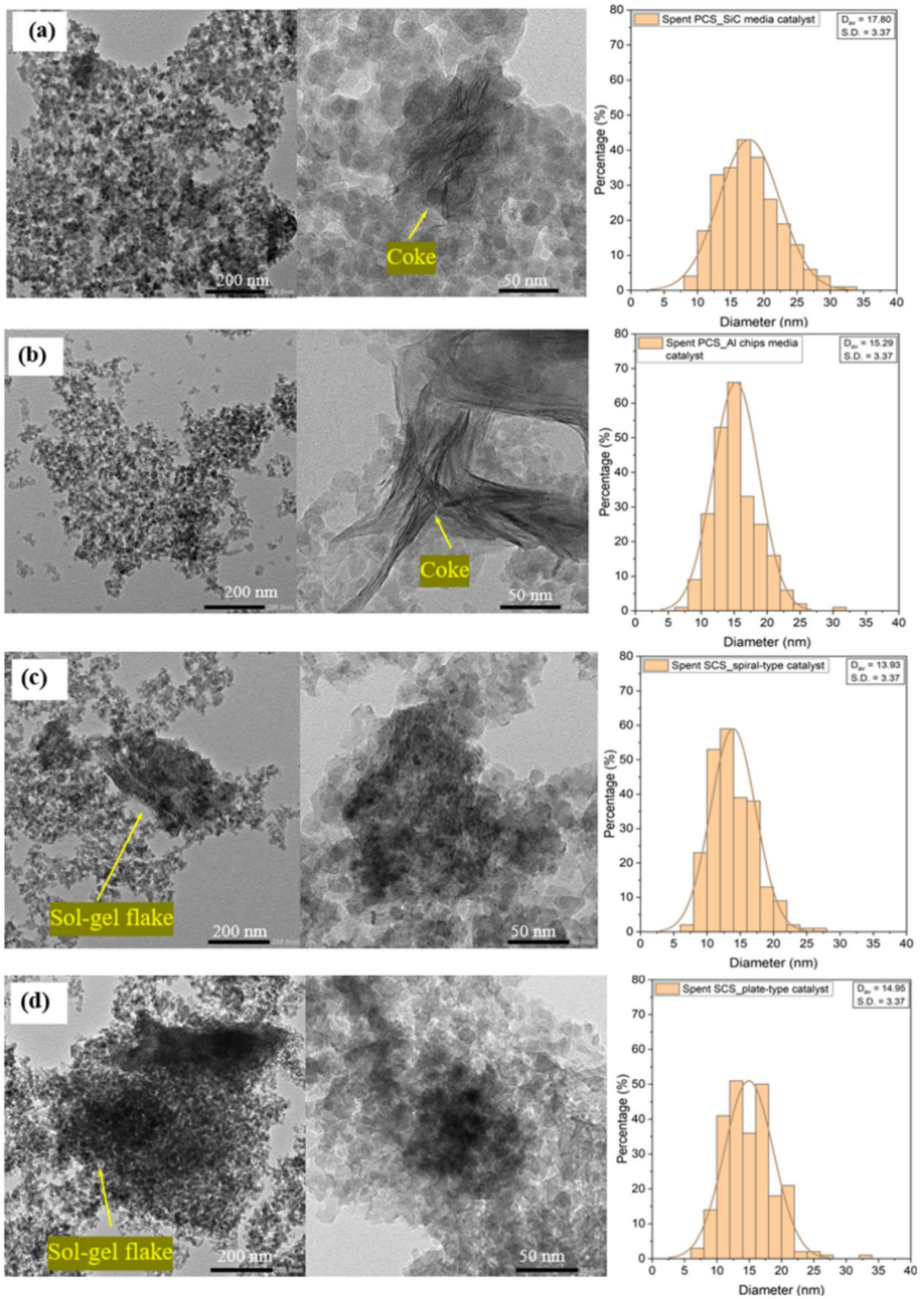
TEM images and particles size distribution of spent catalysts: (**a**) spent PCS_SiC media catalyst, (**b**) spent PCS_Al chips media catalyst, (**c**) spent SCS_spiral-type catalyst and (**d**) spent SCS_plate-type catalyst.

Figure 12 shows TEM images of spent catalysts. The surface morphology of coke was found in both spent PSC catalysts which occurred during FTS reaction^18^. A larger particle size of the spent PSC catalyst resulted from the deposition of carbon species and the metal sintering as the XRD result showed the large particles of Co metal. Meanwhile, the spent structured catalysts exhibited a slight change in particle size. The formed cokes were disordered graphitic carbon and filamentous coke (Fig. 12a,b). These morphologies were influenced by the type of catalyst used, the operating conditions, and the chemical reaction itself^19^. The filamentous coke had a width in the nanometer range and a length of several micrometers. This finding was consistent with the SEM results.

The element composition of catalysts was determined by ICP-OES analysis as shown in Table 1. The fresh SCS catalyst presented a lower cobalt content than the fresh PCS catalyst. This was due to the addition of a binder in the coating methods. Therefore, the sol–gel flakes were observed as shown in Fig. 12c,d. In addition, the fresh and spent SCS catalysts exhibited higher aluminum content due to the presence of aluminum in sol–gel binder. The BET surface area, the total pore volume, and the mean pore diameter of SiO_2_ support and cobalt based catalysts are summarized in Table 1. The SiO_2_ support showed the specific surface area of 164.2 m^2^/g. After loading cobalt onto the support and then reducing it with H_2_, the specific surface area and total pore volume of the fresh PCS catalyst decreased. This indicated that the pores were plugged by cobalt particles. The fresh SCS catalyst exhibited the highest specific surface area due to the presence of alumina on the catalyst, which originated from the binder sol–gel coating process. After FTS reaction, the specific surface area, pore volume, and pore diameter obviously decreased due to the catalyst deactivation as reported in the previous data.

**Fig. 13:**
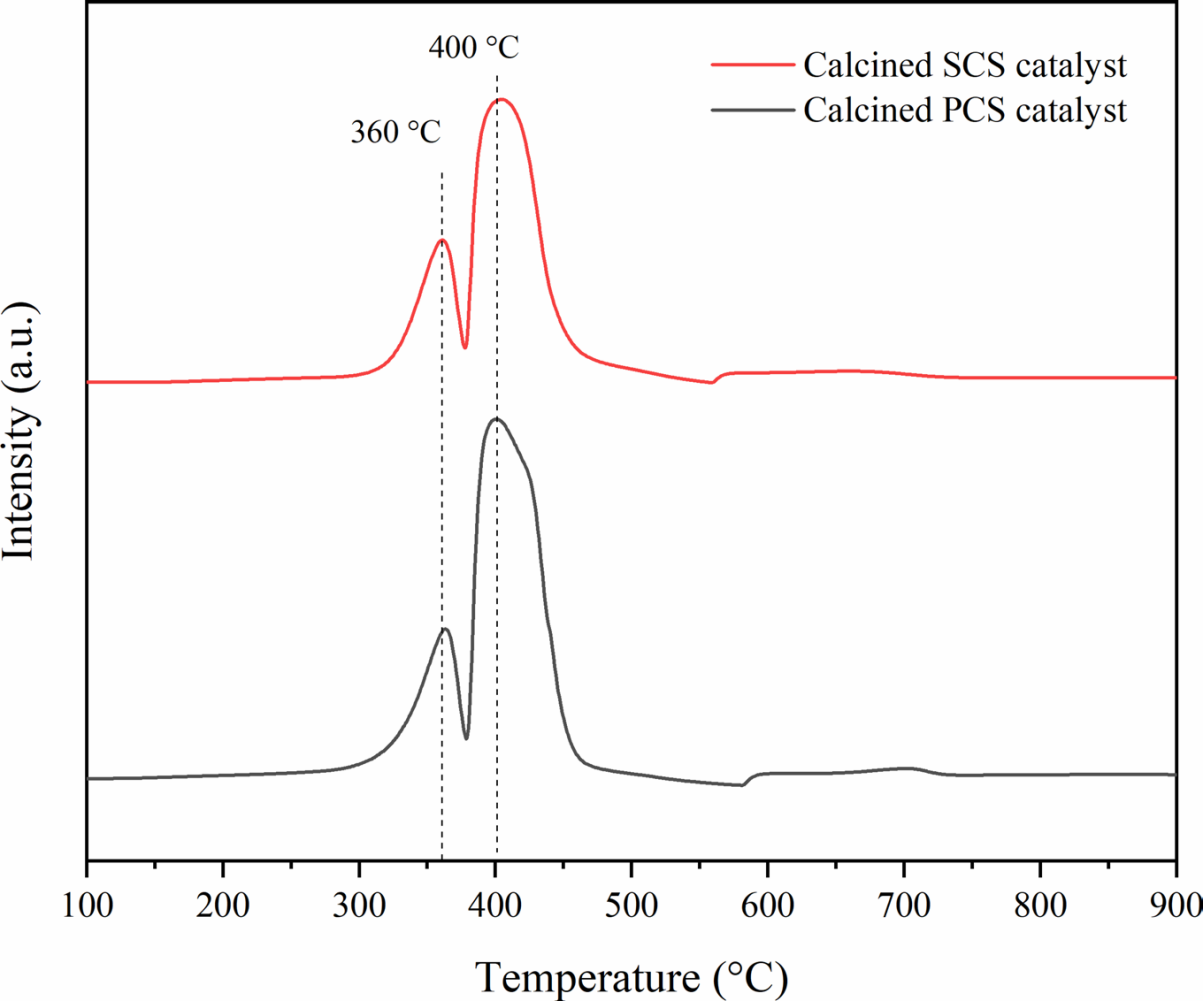
H_2_-TPR profiles of calcined catalysts: structured catalyst and packed bed catalyst.

Figure 13 shows the reduction behavior of both calcined catalysts. The two reduction steps of cobalt oxide can be observed. The first reduction peak attributed to the overlap of Co_3_O_4_ to CoO and then to Co metal^17^. The second reduction peak at high temperature can be assigned to the strong interaction between cobalt and SiO_2_ support^20^. Both calcined catalysts showed a similar pattern of reduction peak. This result confirmed that adding binder to Co/SiO_2_ did not affect the reduction behavior of the catalyst. The broad peak around 550–740 °C indicated the reduction of the cobalt silicate spinel structure.

**Fig. 14:**
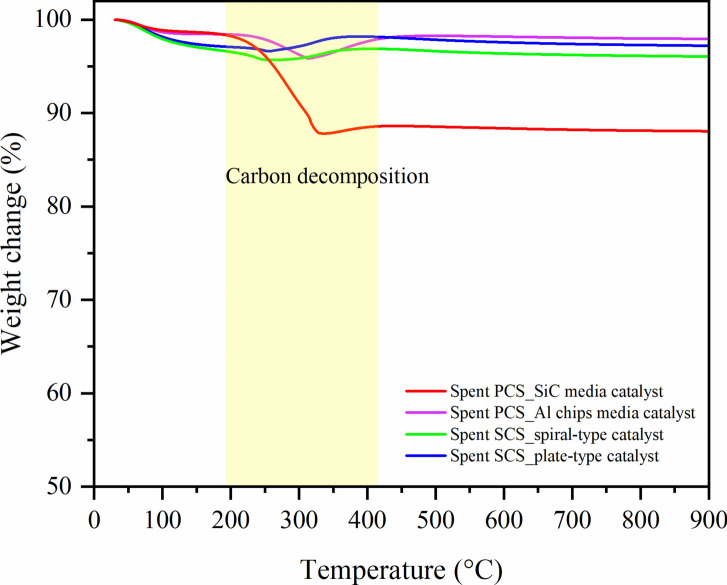
TGA profiles of the spent Co/SiO_2_ catalysts after FTS testing at 220 °C, 20 bar, 8 h.

Figure 14 shows the amount of coke deposits on the spent catalysts determined by the TGA technique. The weight loss observed at temperatures below 180 °C was attributed to the evaporation of moisture and volatile species on catalysts. The temperature between 180 and 330 °C was assigned to the decomposition of amorphous coke^18^. The weight of the catalyst was obviously decreased for all samples because of carbon decomposition. The weight of spent PCS_SiC catalyst was dramatically decreased. This suggested that a large amount of coke had been deposited on the catalyst surface. The results by TGA were in line with the results obtained by SEM and TEM. While the spent SCS catalyst exhibited a slight carbon decomposition and a lower temperature for carbon decomposition. It should be noted that the SCS catalyst decreased coke formation during the FTS reaction. However, the TGA profiles show that slight positive weight changes after 350 °C were observed, ascribed to the oxidation of metallic cobalt in the spent catalysts, resulting in the weight gain of analyzed samples. We found that the mass balance values are low and far from 100% was due to the carbon deposits, as evidenced by TGA analyses.

### Fischer–Tropsch synthesis performance

#### Effect of heat removal by different reactor systems

**Fig. 15:**
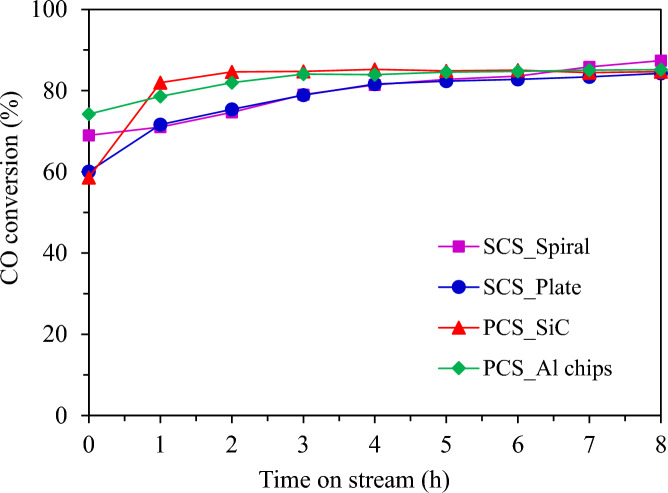
CO conversion from FTS in different reactor systems at 220 °C, 20 bar, 8 h.

Figure 15 shows the CO conversion with time on stream while the FTS catalytic performance and product distributions of the structured catalyst system and the packed bed system are summarized in Table 2. Both catalytic systems showed high CO conversion around 78–82%. The packed bed catalytic system exhibited higher CO conversion and promoted C_5+_ selectivity than the structured catalytic system due to smaller metallic cobalt particles and well dispersion. The fresh PCS catalyst gave the highest cobalt composition in the catalyst as determined by ICP data. This result was possible to influence the catalytic performance of the FTS. The better dispersion of cobalt metal significantly promoted the chain growth probability of hydrocarbons, leading to the formation of heavy hydrocarbons^21^.

**Table 2:** The FTS catalytic performance and product distributions of the structured and the packed bed catalyst system.

Reactor system	CO conv. (%)^a^	Product selectivity (%)^a^				Oil phase selectivity (%)			Solid product^a^ (g)	Gas product (g)	Oil product (g)	Water product (g)	Mass balance^b^ (%)
		CH_4_	CO_2_	C_2_H_6_	C_5+_	C_5-11_	C_12-20_	C_21+_					
SCS_spiral-type catalyst	79.41	7.70	3.50	0.96	87.83	35.38	63.71	0.92	0.23 (2.68 wt%) ^b^	1.69 (19.51wt%)	0.09 (1.04 wt%)	6.67 (76.77 wt%)	62.67
SCS_plate-type catalyst	77.82	8.25	3.63	1.02	87.10	ND	ND	ND	0.18 (2.04 wt%)	1.88 (21.46 wt%)	ND	6.69 (76.50 wt%)	61.05
PCS_SiC media catalyst	81.57	3.01	0.82	0.44	95.73	46.55	53.10	0.36	0.66 (5.58 wt%)	0.54 (4.79 wt%)	1.42 (12.47 wt%)	8.73 (76.88 wt %)	71.37
PCS_Al chips media catalyst	82.47	3.99	1.29	0.64	94.09	65.39	34.32	0.29	0.18 (1.68 wt%)	0.83 (7.64 wt%)	0.71 (6.62 wt%)	9.08 (84.07 wt%)	66.75

*SCS* structured catalytic system, *PCS* packed bed catalytic system. ^a^Calculated from carbon deposits. ^b^Calculated from mass balance, FTS condition: CO/H_2_ = 1/2 molar ratio, T = 220 °C, and P = 20 bar, Time 8 h.

The production of heavy hydrocarbons also accelerated the coke formation on catalyst surface. This is the result of the coke formation, that was clearly observed on the PCS catalyst surface. The structured catalytic system showed higher gas production, especially in CH_4_ and CO_2_ selectivity. This could be attributed to hotspots occurring on the catalyst surface, enhancing the hydrogenation of surface-active carbonized species. The high CO_2_ selectivity was evidently a result of the water–gas shift reaction occurring over the catalyst^22^. From the obtained liquid oil product, all packed bed catalytic systems presented high production. The packed bed catalytic system with SiC media produced the most liquid oil product in this experiment, while the structured catalytic system with plate catalyst did not yield any liquid oil product. Thermal conductive material media (SiC and Al chip) in a packed bed catalytic system show no significant difference in CO conversion and C_5+_ selectivity. However, there are clear differences in oil product distribution. The PCS_SiC media catalyst exhibited the highest selectivity for heavy hydrocarbon (C_12_–C_20_), whereas the PCS_Al media catalyst demonstrated the highest selectivity for light hydrocarbon (C_5_–C_11_). It was found that effective heat-conducting substrates are essential for temperature control in packed bed systems employed in FTS. This can be achieved by using materials with high thermal conductivity. Utilizing materials with high thermal conductivity enables improved temperature control, enhancing the production of larger hydrocarbon product (C_12_–C_20_) at higher temperatures without a significant increase in unwanted methane production^23^. Considering the range of oil products, the structured catalytic system with a spiral-type catalyst obtained greater selectivity for the diesel range, which is the desired product in this research. For water production in FTS, all reactor systems showed a high production yield of water. This can be explained by the increased WGS activity of the catalyst at high CO conversion. Moreover, water is a major byproduct derived from the FT reaction, leading to higher water concentration levels in the total liquid product^24^. The PSC catalyst showed higher water product. This may be a reason for catalyst deactivation through the leaching of active phase and metal sintering, correlated with the decline of cobalt composition as result in ICP data and the large particles of Co metal as the XRD result. Generally, FTS is a highly exothermic reaction that has the potential to induce metal sintering. Metal sintering is considered an irreversible phenomenon in which crystallite migration occurs at high temperatures, and water vapor (under FTS conditions) accelerates the process.

**Fig. 16:**
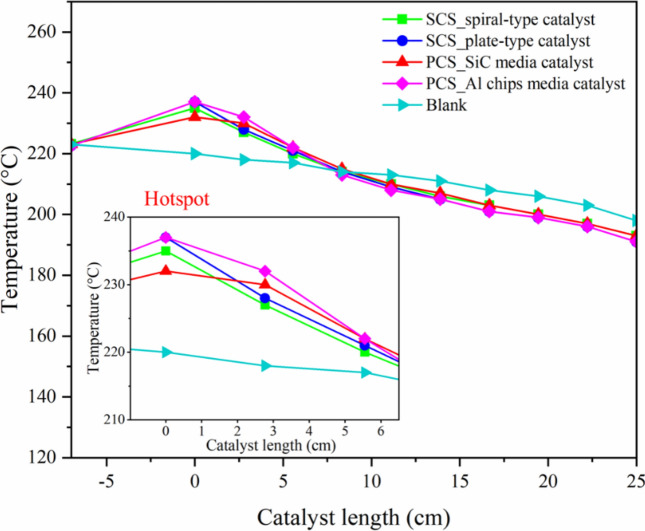
Temperature profile of structured catalytic system and packed bed catalytic system along catalyst bed length.

Figure 16 shows temperature profiles of the structured catalytic system and packed bed catalytic system. The formation of a hotspot was detected by a temperature indicator along the length of the catalyst bed and was presented as a temperature profile. In the case of the structured catalytic system, the temperature profile of the plate type exhibited a higher temperature than that of the spiral-type at the same catalyst length. This suggested that more heat was removed from the catalyst, resulting in increased heat transfer to the system. The SCS_spiral-type catalyst induced a flow pattern in the spinning direction, allowing reactant gases to fully access the catalyst surface. Moreover, the mass transfer limitations are diminished^11^. While the flow pattern of the SCS plate-type catalyst is straight direction, causing the reactant gas to access the catalyst less efficiently than in a spiral-structured catalytic system. Unfortunately, this also affects heat removal^25,26^.

In the event of packed bed catalytic system, both packed bed catalytic system with Al and SiC media exhibited a random flow pattern, resulting in poor heat removal. The spiral structured catalyst can enhance heat and mass transfer in FTS system. Focusing on the development of heat transfer by incorporating a highly thermally conductive material, SiC media exhibited significant improvement in heat transfer due to its high thermal conductivity at 490 W/m·K^27^. Meanwhile, the thermal conductive material of aluminum is 237 W/m.K. This property is essential for the efficient heat removal in packed bed catalytic systems utilizing SiC media. Therefore, the flow pattern of gas through the catalyst system and material media significantly affects heat transfer in the system. However, the structured catalyst is not the most effective method for heat removal in this experimental setup because the media used in this experiment is SiC, which has high thermal conductivity. A spiral-structured catalyst system alone may not be able to overcome this difference.

#### Effect of heat removal by different dilution ratios

**Fig. 17:**
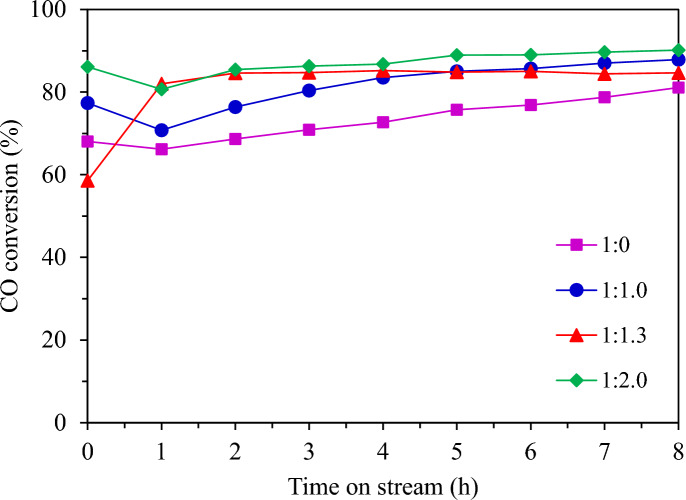
CO conversion from FTS by different dilution ratios at 220 °C, 20 bar, 8 h.

Figure 17 shows the CO conversion with time on stream by different dilution ratios while the FTS catalytic performance and product distributions of granular catalytic system with different dilution ratios of catalyst and SiC are shown in Table 3. The granular catalyst without dilution of SiC media exhibited the lowest CO conversion and promoted methanation. The liquid oil product yield cannot be collected in this system. The increased SiC media ratio in the packed bed granular catalytic system resulted in higher CO conversion and selectivity for C_5+_. The ratio of 1:1.3 presented the highest C_5+_ selectivity and liquid oil product yield. It was remarked that the oil product was in the diesel range.

**Table 3:** The FTS catalytic performance and product distributions of the granular catalytic system with different dilution ratios of catalyst and SiC media.

Dilution ratio	CO conv. (%)	Product selectivity (%)^a^				Oil phase selectivity (%)			Solid product (g)	Gas product (g)	Oil product (g)	Water product (g)	Mass balance^b^ (%)
		CH_4_	CO_2_	C_2_H_6_	C_5+_	C_5-11_	C_12-20_	C_21+_					
1:0.0	73.22	18.96	7.66	1.17	72.20	ND	ND	ND	0.36 (4.45 wt%)	3.59 (44.95 wt%)	ND	4.05 (50.60 wt%)	65.01
1:1.0	81.58	7.21	2.70	0.81	89.28	47.44	51.43	1.13	0.36 (3.46 wt%)	1.42 (13.63 wt%)	0.36 (3.46 wt%)	8.30 (79.45 wt %)	70.42
1:1.3	81.57	3.01	0.82	0.44	95.73	46.55	53.10	0.36	0.66 (5.58 wt%)	0.54 (4.79 wt%)	1.42 (12.47 wt%)	8.73 (76.88 wt %)	71.37
1:2.0	87.47	2.99	1.08	0.42	95.51	71.15	28.72	0.13	0.06 (0.66 wt%)	0.60 (6.84 wt%)	0.55 (6.27 wt%)	7.60 (86.22 wt%)	58.40

^a^Calculated from carbon deposits, ^b^Calculated from mass balance, FTS condition: CO/H_2_ = 1/2 molar ratio, T = 220 °C, P = 20 bar, Time = 8 h, (granular Co/SiO_2_: SiC = 1:0, 1:1.0, 1:1.3, and 1:2.0).

**Fig. 18:**
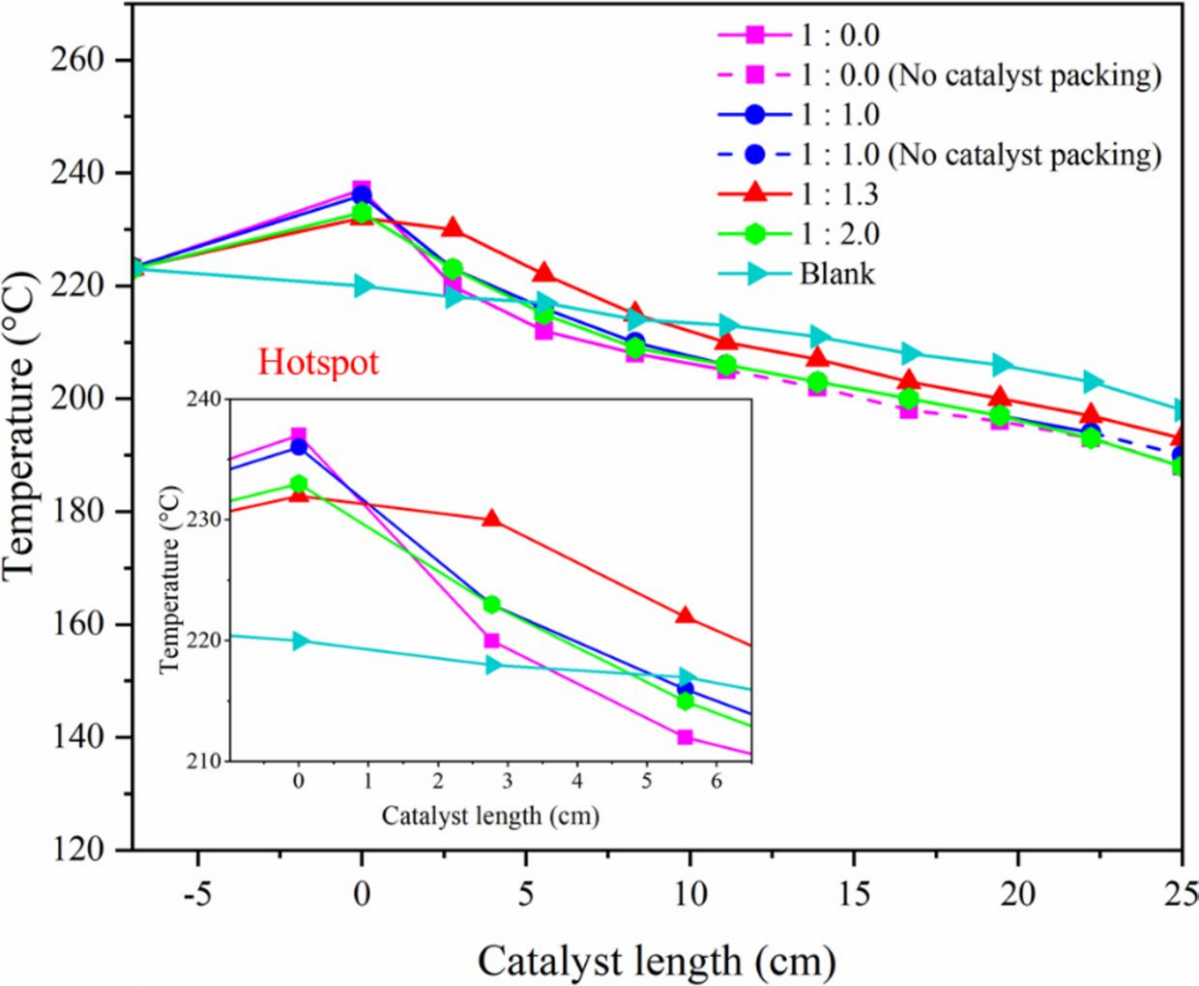
Temperature profile of granular catalytic system with different dilution ratios of catalyst and SiC media.

Figure 18 shows the temperature profile of granular catalytic system with different dilution ratios of catalysts and SiC media. Increasing the SiC ratio from 0 to 1.3 improved heat transfer, but further increasing the SiC ratio to 2.0 decreased heat transfer. Generally, adding more SiC was expected to improve heat removal but it has limitations. It can be observed that the temperature profile of SiC at a ratio of 1.3 is more stable with longer catalyst lengths. Therefore, this dilution ratio is appropriate to develop heat removal for Fischer–Tropsch synthesis to produce diesel range production.

#### Optimization condition

##### Effect of reaction temperature for FTS

**Fig. 19:**
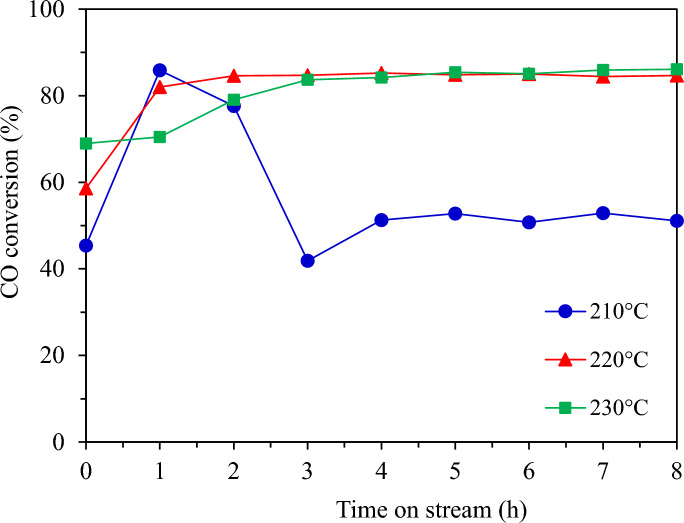
CO conversion from FTS at different reaction temperatures at 20 bar, 8 h.

**Fig. 20:**
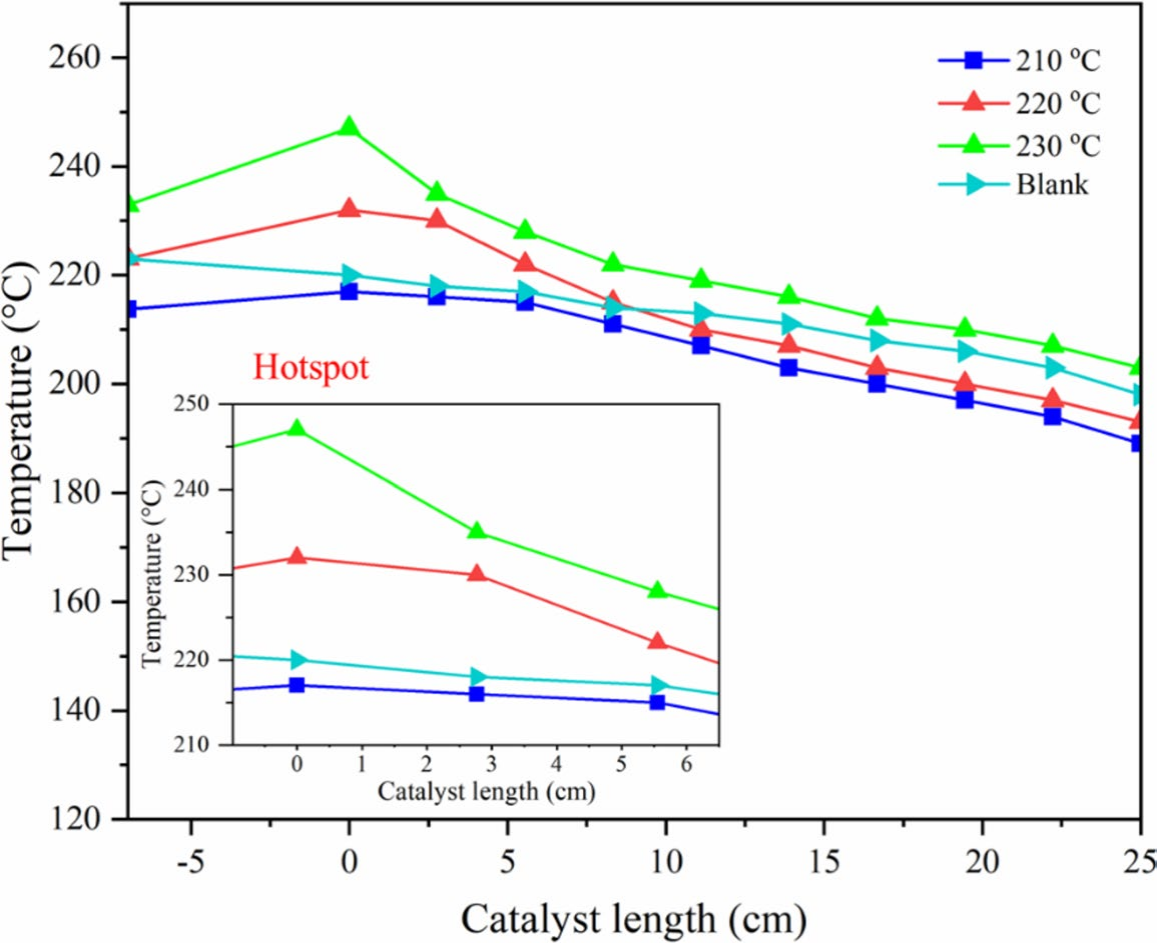
Temperature profile of packed bed granular catalytic system with different temperatures.

The effects of temperature and cobalt metal loading were investigated to optimize the catalyst and reaction condition. First, the FTS catalytic performance and product distributions of packed bed granular catalytic system with different temperatures are listed in Table 4, while Fig. 19 shows the CO conversion by different reaction temperatures. Increasing the reaction temperature from 210 to 220 °C enhanced CO conversion, selectivity of C_5+_ and liquid oil products, but it decreased the oil products in the diesel range. At 230 °C, CO conversion hardly changed, but the products in the diesel range dramatically dropped. It is well known that FTS is a strong exothermic reaction, and the increase of temperature is not favorable to long-chain hydrocarbons^28^. Moreover, hotspots easily occurred on the catalyst surface at this temperature as shown in Fig. 20. The reaction temperature at 220 °C showed the highest CO conversion and total oil products. Considering the total liquid products (oil and water), this temperature produced the highest oil/water ratio. It was implied that the reaction at this temperature promoted the hydrocarbon production pathways. Thus, the optimum temperature is 220 °C which enhances the catalytic performance for FTS.

**Table 4:** The FTS catalytic performance and product distributions of the packed bed system with different temperatures.

Temperature (°C)	CO conv. (%)	Product selectivity (%)				Oil phase selectivity (%)			Solid product^a^ (g)	Gas product (g)	Oil product (g)	Water product (g)	Mass balance^b^ (%)
		CH_4_	CO_2_	C_2_H_6_	C_5+_	C_5–11_	C_12–20_	C_21+_					
210	56.62	7.46	1.79	0.94	89.80	41.40	58.56	0.04	0.17 (2.34 wt%)	0.78 (10.70 wt%)	0.23 (3.09 wt%)	6.13 (83.87 wt%)	76.89
220	81.57	3.01	0.82	0.44	95.73	46.55	53.10	0.36	0.66 (5.58 wt%)	0.54 (4.79 wt%)	1.42 (12.47 wt%)	8.73 (76.88 wt %)	71.37
230	80.99	5.07	2.02	0.63	92.28	63.17	36.37	0.47	0.19 (1.91 wt%)	1.09 (10.66 wt%)	0.53 (5.23 wt%)	8.37 (82.20 wt%)	66.94

^a^Calculated from carbon deposits, ^b^Calculated from mass balance, FTS condition: CO/H_2_ = 1/2 molar ratio, T = 210–230 °C, P = 20 bar, Time = 8 h, (granular Co/SiO_2_:SiC = 1:1.3).

##### Effect of percent loading of cobalt in catalyst

**Fig. 21:**
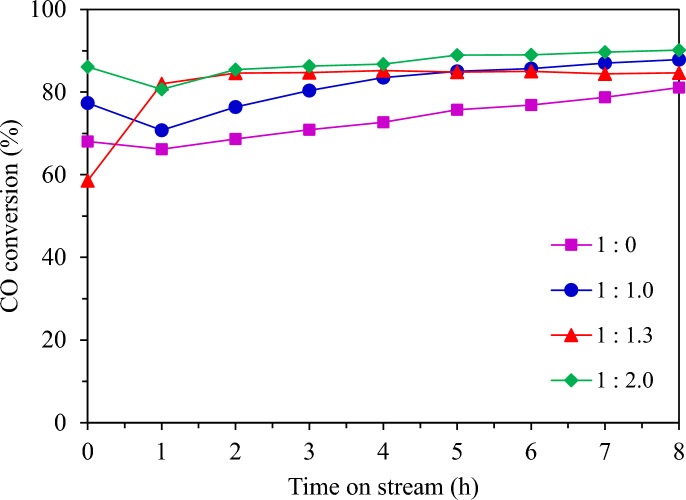
CO conversion from FTS at different cobalt loadings at 220 °C, 20 bar, 8 h.

The various percentages of cobalt supported on SiO_2_ were investigated, and the results are summarized in Table 5. An increase in cobalt loading enhanced CO conversion and products in the diesel range as shown in Fig. 21. It was generally assumed that a higher cobalt loading resulted in a higher number of active cobalt sites and a larger cobalt particle size, which promoted strong CO adsorption, resulting in an increase in CO conversion and the formation of long-chain hydrocarbon^29^. This result was possible to influence the catalytic performance of the FTS. The 20Co/SiO_2_ showed the highest CO conversion and products in the diesel range. Moreover, it showed lower CO_2_ production related to a lower WGS reaction. Thus, the 20Co/SiO_2_ is suitable for this experimental setup.

**Table 5:** The FTS catalytic performance and product distributions of Co/SiO_2_ with different cobalt loadings.

Temperature (°C)	CO conv. (%)	Product Selectivity (%)				Oil phase selectivity (%)			Solid product^a^ (g)	Gas product (g)	Oil product (g)	Water product (g)	Mass balance^b^ (%)
		CH_4_	CO_2_	C_2_H_6_	C_5+_	C_5-11_	C_12-20_	C_21+_					
15Co/SiO_2_	77.58	3.27	1.04	0.52	95.17	64.86	34.90	0.24	0.50 (4.20 wt%)^b^	0.66 (5.58 wt%)	0.75 (6.14 wt%)	9.91 (84.08 wt%)	72.75
20Co/SiO_2_	81.57	3.01	0.82	0.44	95.73	46.55	53.10	0.36	0.66 (5.58 wt%)	0.54 (4.79 wt%)	1.42 (12.47 wt%)	8.73 (76.88 wt %)	71.37
30Co/SiO_2_	81.64	6.26	2.74	0.70	90.30	42.69	56.27	0.74	0.39 (3.85 wt%)	1.42 (14.06 wt%)	0.35 (3.48 wt%)	7.93 (78.62 wt%)	65.72

^a^Calculated from carbon deposits, ^b^Calculated from mass balance, FTS condition: CO/H_2_ = 1/2 molar ratio, T = 220 °C, P = 20 bar, Time = 8 h, (granular Co/SiO_2_:SiC = 1:1.3).

## Conclusion

In this research work, the catalytic performance of Co/SiO_2_ catalysts using different catalytic systems on Fischer–Tropsch synthesis (FTS) was investigated. The packed bed catalytic system with SiC as heat removal material showed the highest oil production due to the high thermal conductivity of SiC. While the structured catalytic system with spiral-type could enhance the product selectivity in desired range of green diesel but the oil production yield was undesirable. In terms of heat transfer effectiveness, the form of structured catalysts, dilution media type, and ratio of dilution media significantly improve the heat transfer in FTS system. The hotspot formation of SCS_spiral-type catalyst significantly decreased, compared to the plate-type and Al chip catalysts. This was due to heat transfer improved by the reactant gas flowing in contact with the catalyst surface in the spiral shape. Unfortunately, the spiral-type structured catalytic system has lower conductivity, compared to the thermal conductivity of SiC. Therefore, the packed bed catalytic system with SiC media showed the best heat removal and oil production. The optimum metal loading and conditions for the packed bed catalytic system are 20 wt% Co loading, a dilution ratio of 1:1.3 (catalyst to SiC), and a reaction temperature of 220 °C, respectively.

## Data Availability

All data related to the finding of this study are accessible upon request from the corresponding author Sakhon Ratchahat.
